# Cell Types and Synapses Expressing the SNARE Complex Regulating Proteins Complexin 1 and Complexin 2 in Mammalian Retina

**DOI:** 10.3390/ijms22158131

**Published:** 2021-07-29

**Authors:** Uwe Thorsten Lux, Johanna Ehrenberg, Anneka Joachimsthaler, Jenny Atorf, Bianca Pircher, Kerstin Reim, Jan Kremers, Andreas Gießl, Johann Helmut Brandstätter

**Affiliations:** 1 Division of Animal Physiology, Department of Biology, Friedrich-Alexander-Universität Erlangen-Nürnberg, 91058 Erlangen, Germany; uwe.lux@fau.de (U.T.L.); johanna.ehrenberg@fluidic.com (J.E.); bianca.pircher@fau.de (B.P.); 2 Department of Ophthalmology, University Hospital Erlangen, Friedrich-Alexander-Universität Erlangen-Nürnberg, 91054 Erlangen, Germany; anneka.joachimsthaler@uk-erlangen.de (A.J.); jenny.atorf@klinikum-karlsruhe.de (J.A.); jan.kremers@uk-erlangen.de (J.K.); andreas.giessl@uk-erlangen.de (A.G.); 3 Department of Molecular Neurobiology, Max Planck Institute of Experimental Medicine, 37075 Göttingen, Germany; reim@em.mpg.de

**Keywords:** complexin 1, complexin 2, conventional chemical synapses, amacrine cells, ganglion cells, horizontal cells, retina

## Abstract

Complexins (Cplxs) 1 to 4 are components of the presynaptic compartment of chemical synapses where they regulate important steps in synaptic vesicle exocytosis. In the retina, all four Cplxs are present, and while we know a lot about Cplxs 3 and 4, little is known about Cplxs 1 and 2. Here, we performed in situ hybridization experiments and bioinformatics and exploited Cplx 1 and Cplx 2 single-knockout mice combined with immunocytochemistry and light microscopy to characterize in detail the cell type and synapse-specific distribution of Cplx 1 and Cplx 2. We found that Cplx 2 and not Cplx 1 is the main isoform expressed in normal and displaced amacrine cells and ganglion cells in mouse retinae and that amacrine cells seem to operate with a single Cplx isoform at their conventional chemical synapses. Surprising was the finding that retinal function, determined with electroretinographic recordings, was altered in Cplx 1 but not Cplx 2 single-knockout mice. In summary, the results provide an important basis for future studies on the function of Cplxs 1 and 2 in the processing of visual signals in the mammalian retina.

## 1. Introduction

At most neuronal synapses, tripartite SNARE complexes, composed of the vesicle SNARE synaptobrevin on the synaptic vesicle membrane and the target SNAREs SNAP-25 and syntaxin on the plasma membrane, drive the Ca^2+^-triggered fusion of synaptic vesicles, one of the most tightly regulated membrane fusion reactions [1]. SNARE complex regulating proteins precisely control the speed and temporal precision of SNARE complex assembly and synaptic vesicle fusion. Important SNARE complex regulators are the complexins (Cplxs)—small, soluble proteins that bind to the SNARE complexes and either stabilize them in a highly fusogenic state or arrest them to prevent fusion [2]. In mammals, four Cplx isoforms exist, which are subdivided into two subfamilies: Cplx 1/2 and Cplx 3/4 [3,4]. The two subfamilies share limited homology and they differ structurally by the presence of a C-terminal CAAX farnesylation motif in Cplxs 3 and 4, which regulates their synaptic targeting and membrane association [4]. While almost all neuron types express Cplx 1 and/or Cplx 2, Cplx 3 is only weakly and Cplx 4 is not expressed in the brain. Interestingly, in the mammalian retina, which is part of the brain, all four Cplxs are present [4].

In the retinal network of sensory and higher-order neurons, visual signals are processed at structurally and functionally distinct types of chemical synapses: the phasically transmitting conventional chemical synapses of amacrine cells and the tonically transmitting ribbon synapses of photoreceptors and bipolar cells [5]. As a special structural and functional adaptation to the high rates of tonic neurotransmitter release, retinal ribbon synapses are equipped with a presynaptic electron-dense, plate-like organelle—the synaptic ribbon. Especially, the photoreceptor ribbons tether hundreds of release-ready synaptic vesicles in close proximity to voltage-gated Ca^2+^ channels at the active zone, accommodating the high rates of neurotransmitter release [5,6,7,8]. Special molecular constituents, such as Cplxs 3 and 4, are suggested to contribute to the unique release efficacy of photoreceptor ribbon synapses. We and others have shown that Cplxs 3 and 4 exert different regulatory effects on synaptic vesicle exocytosis at retinal ribbon synapses by suppressing tonic, spontaneous neurotransmitter release and facilitating depolarization-evoked release [4,9,10,11]. Furthermore, Cplxs 3 and 4 play a regulatory role in the adaptation-dependent availability of synaptic vesicles at mouse photoreceptor ribbon synapses [10]. Because of the high homology in the central α-helical region within the mammalian Cplx family, it is suggested that analogous to Cplxs 1 and 2 in conventional chemical synapses, Cplxs 3 and 4 in ribbon synapses express their function by binding to and regulating the SNARE complexes [9,12,13].

Unlike Cplxs 3 and 4, we know little about Cplxs 1 and 2 in the retina. A reason is the lack of specific antibodies against the two Cplxs due to their high amino acid sequence homology (80% identity) [4]. Employing Cplx 1 and Cplx 2 single-knockout (KO) mice [14], we here provide a comprehensive survey of the cell type and synapse-specific expression of Cplxs 1 and 2 in mouse retina.

## 2. Results

### 2.1. Cplx 2 but Not Cplx 1 Is Expressed by Cells in the Inner Nuclear Layer in Mouse Retina

**Figure 1:**
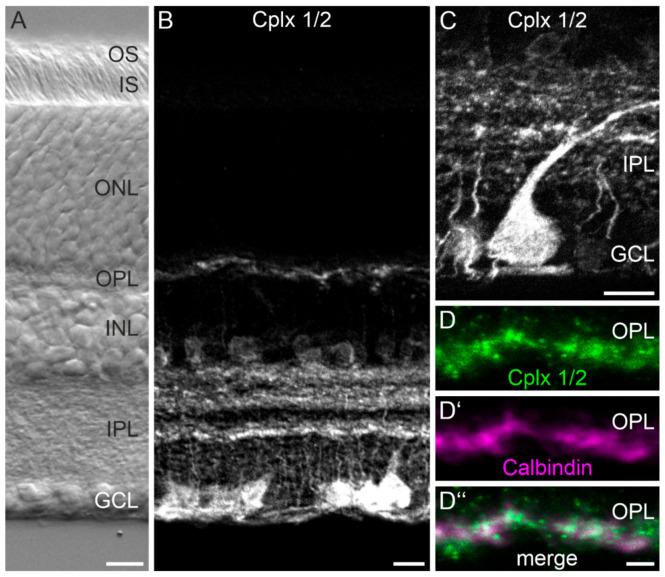
Distribution of Cplx 1/2 in mouse retina. (**A**) Nomarski micrograph of a vertical vibratome section through a mouse retina showing the different retinal layers. (**B**,**C**) Fluorescence micrographs of vertical vibratome sections through mouse retinae stained with an anti-Cplx 1/2 antibody. A higher-power view of Cplx 1/2 staining in the inner plexiform layer (IPL) and ganglion cell layer (GCL) is shown in (**C**). (**D**–**D″**) Fluorescence micrographs showing a part of the outer plexiform layer (OPL) double labeled for Cplx 1/2 and calbindin to mark the horizontal cells. OS, outer segments; IS, inner segments; ONL, outer nuclear layer; INL, inner nuclear layer. Scale bar = 10 µm in (**A**–**C**) and 2 µm in (**D″**) for (**D**–**D″**).

We first labeled vertical vibratome sections from wildtype (WT) mouse retinae using an anti-Cplx 1/2 antibody that recognizes both isoforms (Figure 1). Vibratome sections were employed because tissue preservation and thus cellular morphology is significantly improved compared to cryostat sections. The anti-Cplx 1/2 antibody labeled amacrine cells with their somata in the inner part of the inner nuclear layer (INL) adjacent to the inner plexiform layer (IPL) and ganglion cells and displaced amacrine cells with their somata in the ganglion cell layer (GCL) (Figure 1A,B). The Cplx 1/2-positive processes and dendrites of the amacrine and ganglion cells, respectively, stratified in the IPL in three distinct broad strata (Figure 1A,B). Figure 1C shows a higher-power view of a Cplx 1/2-labeled cell in the GCL. Due to the large soma size, it is most likely a ganglion cell, which extends a stout dendrite into the OFF sublamina of the IPL. The weak Cplx 1/2 signal in the outer plexiform layer (OPL; Figure 1B) is present in processes of horizontal cells as seen in the double labeling experiments of Cplx 1/2 with the horizontal cell marker calbindin (Figure 1D–D″). The results shown in Figure 1 agree well with those of our previously published study [4].

**Figure 2:**
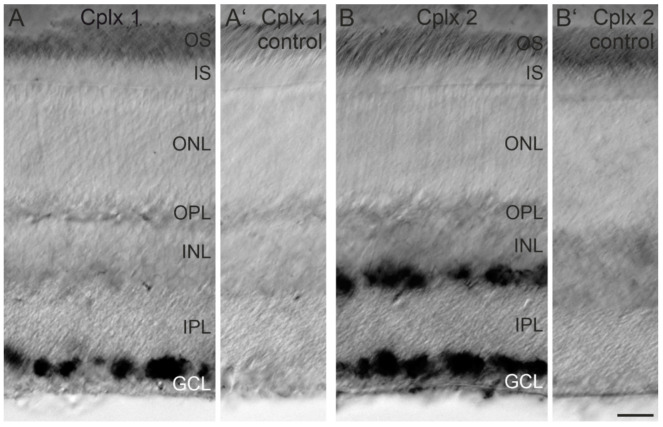
Expression of Cplx 1 and Cplx 2 mRNA in the inner nuclear layer (INL) and ganglion cell layer (GCL) of mouse retina. (**A**) Strong in situ hybridization signal for Cplx 1 mRNA in the GCL. (**A′**) No signal for Cplx 1 with the sense control. (**B**) Strong in situ hybridization signal for Cplx 2 mRNA in the INL and GCL. (**B′**) No signal for Cplx 2 with the sense control. OS, outer segments; IS, inner segments; ONL, outer nuclear layer; OPL, outer plexiform layer; IPL, inner plexiform layer. Scale bar = 20 µm in (**B′**) for (**A**–**B′**).

Because the anti-Cplx 1/2 antibody does not differentiate between Cplx 1 and Cplx 2, we next performed in situ hybridization experiments, allowing a first rough cell-type-specific assignment of the two Cplx isoforms in mouse retina (Figure 2). In vertical vibratome sections, only cells in the GCL displayed a strong signal for the Cplx 1 transcript, suggesting the exclusive expression of Cplx 1 by ganglion cells and/or displaced amacrine cells (Figure 2A). In contrast, a strong signal for the Cplx 2 transcript was present both in cells in the GCL and the INL close to the IPL, suggesting the expression of Cplx 2 in ganglion cells and/or displaced amacrine cells in the GCL and in amacrine cells in the INL (Figure 2B).

### 2.2. Retinal Structure Is Unaltered in Cplx 1 and Cplx 2 KO Mice

**Figure 3:**
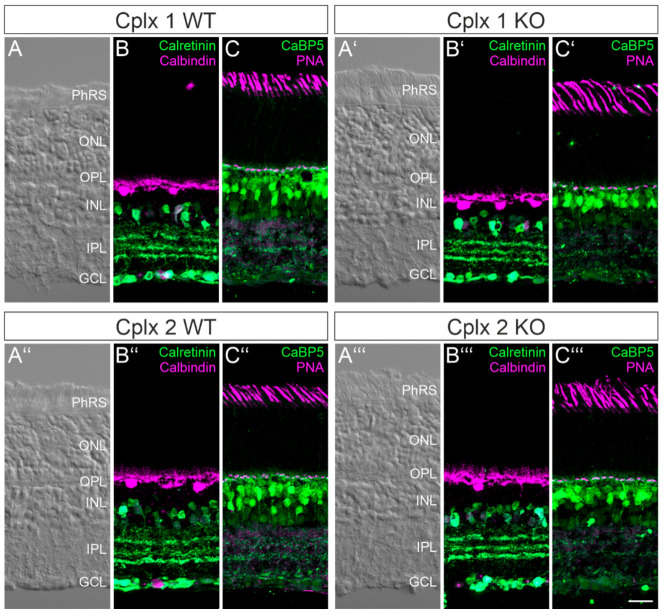
Comparison of the retinal structure between wildtype (WT) and Cplx 1 and Cplx 2 knockout (KO) mice. (**A**–**A‴**) Nomarski micrographs of vertical cryostat sections through the retinae of WT and Cplx 1 and Cplx 2 KO mice. (**B**–**B‴**) Fluorescence micrographs of vertical cryostat sections through the retinae of WT and Cplx 1 and Cplx 2 KO mice double labeled for amacrine and ganglion cells (anti-calretinin) and horizontal cells (anti-calbindin). (**C**–**C‴**) Fluorescence micrographs of vertical cryostat sections through the retinae of WT and Cplx 1 and Cplx 2 KO mice double labeled for bipolar cells (anti-CaBP5) and cone photoreceptors (PNA). PhRS, photoreceptor segments; ONL, outer nuclear layer; OPL, outer plexiform layer; INL, inner nuclear layer; IPL, inner plexiform layer; GCL, ganglion cell layer. Scale bar = 20 µm in (**C‴**) for (**A**–**C‴**).

Because of the lack of antibodies, which separately and specifically detect Cplxs 1 or 2, we decided to use Cplx 1 and Cplx 2 KO mice as a tool to immunocytochemically analyze the individual expression of Cplxs 1 and 2 in amacrine and ganglion cells in mouse retina. Beforehand, however, we examined whether retinal structure is altered in the Cplx 1 and Cplx 2 KO mice. A comparison of Nomarski micrographs of the vertical retinal cryostat sections of Cplx 1 and Cplx 2 KO mice with their WT littermates showed that lack of Cplx 1 or Cplx 2 did not affect the overall structure of the retina with respect to thickness and layering (Figure 3A–A‴). For a more detailed analysis of neuronal morphology, we labeled the main classes of the retinal neurons: amacrine and ganglion cells (anti-calretinin [15]; Figure 3B–B‴), horizontal cells (anti-calbindin [16]; Figure 3B–B‴), rod and cone bipolar cells (anti-CaBP5 [17]; Figure 3C–C‴) and cone photoreceptors (rhodamine-coupled peanut agglutinin [18]; Figure 3C–C‴). There was no apparent difference in the morphology and the synaptic stratification patterns of the various neuron types between WT and Cplx 1 and Cplx 2 KO mice (Figure 3).

**Figure 4:**
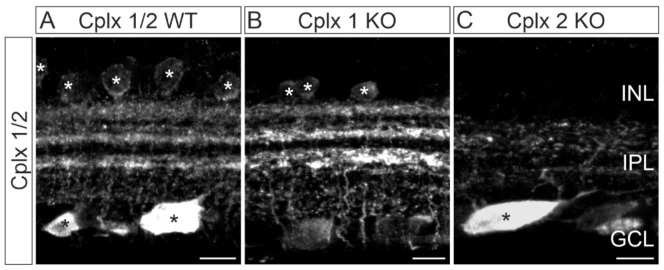
Distribution of Cplx 1/2 in wildtype (WT) and in Cplx 1 and Cplx 2 knockout (KO) mouse retinae. (**A**–**C**) Fluorescence micrographs of vertical vibratome sections through WT and Cplx 1 and Cplx 2 KO mouse retinae stained with an anti-Cplx 1/2 antibody. White asterisks in (**A**,**B**) mark labeled amacrine cell somata in the inner nuclear layer (INL); black asterisks in (**A**,**C**) mark strongly labeled ganglion cell somata in the ganglion cell layer (GCL). IPL, inner plexiform layer. Scale bars = 10 µm.

The morphological data document that Cplx 1 and Cplx 2 KO mice are suitable to perform a detailed analysis of the individual expression pattern of Cplx 1 and Cplx 2 in mouse retina. This notion is also supported by the following results. We repeated our labeling experiments with the anti-Cplx 1/2 antibody and compared stained vertical vibratome sections of WT retinae with those derived from Cplx 1 and Cplx 2 KO mice (Figure 4). Cplx 1/2 staining in WT and Cplx 1 KO retinae hardly differed from each other (Figure 4A,B). In contrast, Cplx 2 KO retinae lacked staining of amacrine cells in the INL and of the three distinct strata of amacrine and ganglion cell processes in the IPL (Figure 4A,C). These results are in good agreement with the in situ hybridization data and confirm our previous assumption that between Cplxs 1 and 2, Cplx 2 is the predominant Cplx isoform in amacrine cells of mouse retina.

### 2.3. Retinal Function Is Altered in Cplx 1 but Not in Cplx 2 KO Mice

**Figure 5:**
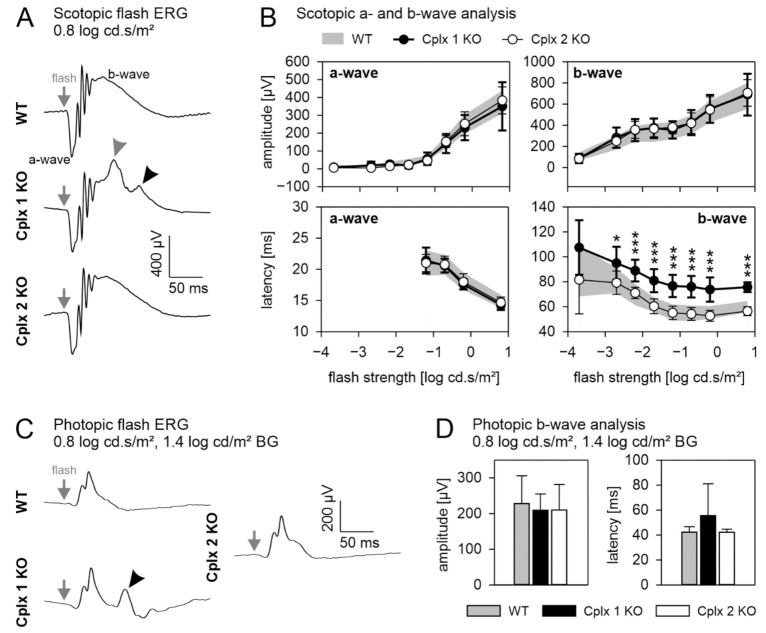
Flash electroretinographic recordings (ERG) of wildtype (WT) and Cplx 1 and Cplx 2 knockout (KO) mice. (**A**) Comparison of the mean ERG responses to a scotopic flash of 0.8 log cd × s/m^2^ between WT and Cplx 1 and Cplx 2 KO mice. Gray arrows indicate flash onset. The black arrowhead highlights the abnormal second peak on the descending part of the b-wave; the gray arrowhead highlights the additional shoulder riding on the peak of the b-wave in Cplx 1 KO mice. (**B**) Amplitude and latency of the scotopic a- and b-wave. WT range is indicated by gray area; Cplx 1 KO, closed symbols; Cplx 2 KO, open symbols. Values are shown as the mean ± SD. Statistical significance is indicated by black asterisks (* *p* < 0.05; *** *p* < 0.001; two-sided *t*-test). Bonferroni-correction factor for multiple testing was 8 for all *p*-values. (**C**) Comparison of the mean ERG responses to a photopic flash of 0.8 log cd × s/m^2^ upon a white background of 1.4 log cd/m^2^ between the WT and Cplx 1 and Cplx 2 KO mice. Gray arrows indicate flash onset. The black arrowhead highlights the abnormal second peak on the descending part of the b-wave in Cplx 1 KO mice. (**D**) Amplitude and latency of the photopic b-wave for WT (gray bar), Cplx 1 KO (black bar) and Cplx 2 KO (white bar). Values are shown as the mean ± SD. Number of animals for scotopic and photopic ERG (WT, *n* = 16; Cplx 1 KO *n* = 7; Cplx 2 KO, *n* = 7).

In addition to our morphological analysis, we examined whether the loss of Cplx 1 or Cplx 2 affects retinal function. We performed electroretinographic (ERG) recordings under scotopic (dark adapted, rod photoreceptor and combined rod-cone photoreceptor mediated) and photopic (light adapted, cone photoreceptor mediated) conditions and compared the results between WT and Cplx 1 and Cplx 2 KO mice (Figure 5). We analyzed the amplitudes and latencies of the a- and b-wave, and the oscillatory potentials (OPs). Under scotopic conditions at a flash strength of 0.8 log cd × s/m^2^, all three mouse lines displayed a typical ERG response containing the negative a-wave, the positive b-wave and the OPs on the rising flank of the b-wave (Figure 5A). Cplx 1 KO mice, however, showed an additional peak of unknown origin on the descending flank of the b-wave (black arrowhead, Figure 5A) and an additional shoulder riding on the peak of the b-wave (gray arrowhead, Figure 5A). Figure 5B summarizes the measured mean amplitudes and latencies of the scotopic a- and b-wave at all measured flash strengths. The amplitudes of the scotopic a- and b-wave and the latencies of the a-wave did not differ between the Cplx 1 KO and Cplx 2 KO mice and their WT controls. Note that the a-wave latency is only shown for the four highest flash strengths where a prominent and reliably measurable a-wave was recorded (Figure 5B). The latencies of the b-wave of Cplx 2 KO mice were comparable to those measured in WT controls, while the b-wave latencies in Cplx 1 KO mice were significantly delayed (Figure 5B). This increased latency is most probably caused by the interference with the abovementioned shoulder that occurs closely to the peak of the b-wave. OPs did not differ between the three mouse lines (data not shown).

Under photopic conditions at a flash strength of 0.8 log cd × s/m^2^ upon a rod photoreceptor saturating 1.4 log cd/m^2^ background light, all three mouse lines displayed a typical ERG response, which is dominated by the b-wave (Figure 5C). As for the scotopic ERG, the Cplx 1 KO mice showed an additional peak in the descending flank of the b-wave (black arrowhead, Figure 5C). Since the additional response components on the descending flank of the b-wave (black arrowhead, Figure 5A,C) in the Cplx 1 mouse in scotopic and photopic conditions have similar delay times, we consider them having identical origins. The measured mean amplitudes and latencies of the photopic b-wave did not differ between the Cplx 1 KO and Cplx 2 KO mice and their WT controls (Figure 5D).

### 2.4. Cplx 2 Expressing Amacrine Cell Types in Mouse Retina

**Figure 6:**
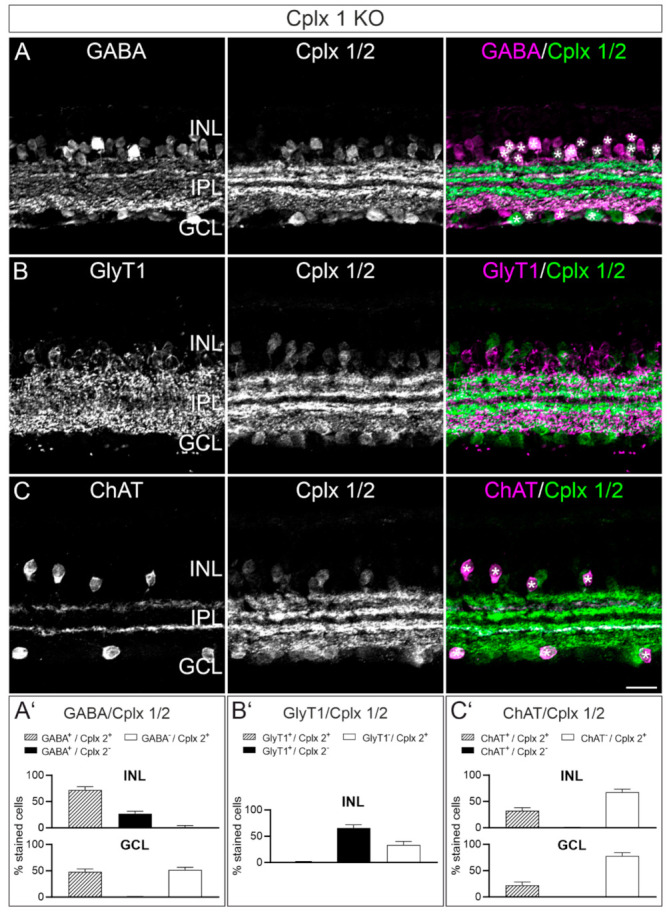
Expression of Cplx 2 in different amacrine cell types in mouse retinae. (**A**–**C**) Fluorescence micrographs of vertical cryostat sections through retinae of Cplx 1 knockout (KO) mice double labeled with antibodies against Cplx 1/2 and the amacrine cell markers γ-aminobutyric acid (GABA; (**A**)), glycine transporter 1 (GlyT1; (**B**)) and choline acetyltransferase (ChAT; (**C**)). White asterisks mark the colocalization of Cplx 2 with GABA (**A**) and ChAT (**C**) in cell somata in the inner nuclear layer (INL) and ganglion cell layer (GCL). No colocalization of Cplx 2 and GlyT1 (**B**). (**A′**–**C′**) Quantification of stained cell somata in the INL and GCL. Sum of all stained cell somata = 100%; number of analyzed cells for (**A′**) 674 (INL) and 536 (GCL); (**B′**) 731 (INL); and (**C′**) 333 (INL) and 442 (GCL). Bar graphs display the mean percentages ± SD in three animals per double labeling experiment. IPL, inner plexiform layer. Scale bar = 20 µm in (**C**) for (**A**–**C**).

**Figure 7:**
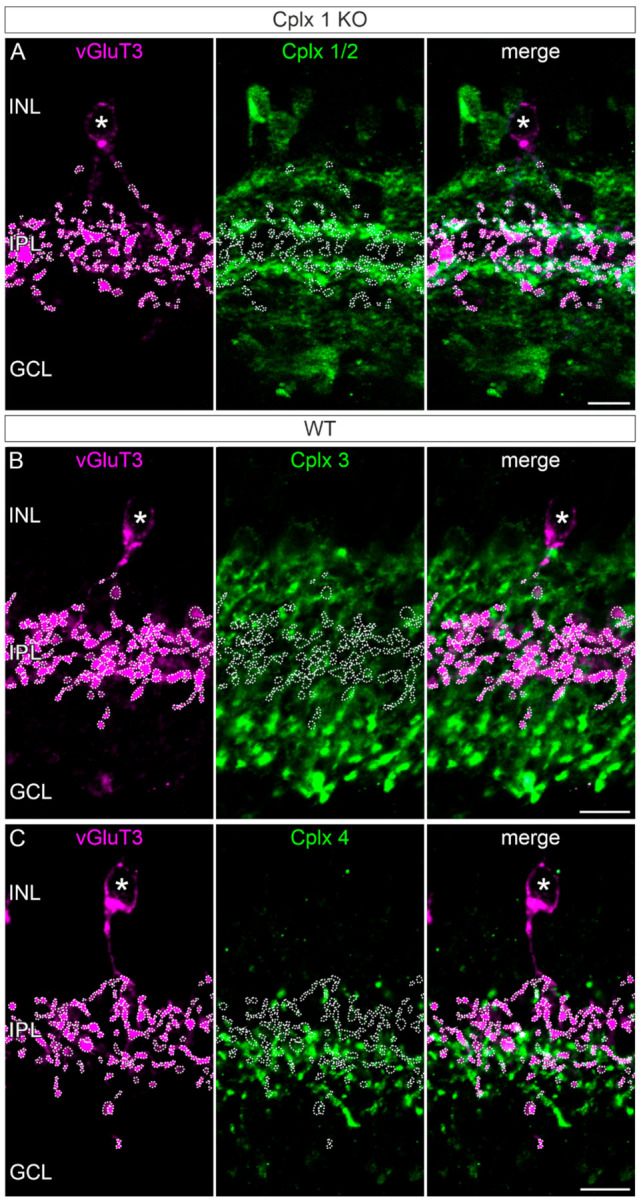
Absence of Cplxs 2 to 4 from vGluT3 amacrine cells in mouse retinae. Fluorescence micrographs of vertical cryostat sections through retinae of Cplx 1 knockout (KO) and wildtype (WT) mice double labeled with antibodies against the vGluT3 (glutamatergic amacrine cells) and (**A**) Cplx 1/2, (**B**) Cplx 3 and (**C**) Cplx 4. The white asterisks mark vGluT3 amacrine cell somata in the inner nuclear layer (INL); the white dotted lines outline the vGluT3 terminals. IPL, inner plexiform layer; GCL, ganglion cell layer. Scale bars = 10 µm.

Between Cplxs 1 and 2, Cplx 2 is the prevailing isoform in amacrine cells in mouse retinae (Figure 2 and Figure 4). Amacrine cells are the most heterogeneous class of retinal interneurons, with over sixty molecularly identified amacrine cell types in the mouse retina [19,20]. To identify amacrine cells that express Cplx 2, we double labeled vertical retinal cryostat sections of Cplx 1 KO mice with the anti-Cplx 1/2 antibody in combination with markers for different amacrine cell types; i.e., antibodies against retinal neurotransmitters and neurotransmitter transporters (Figure 6 and Figure 7).

GABAergic amacrine cells: γ-aminobutyric acid (GABA) is one of the major inhibitory neurotransmitters in the mammalian retina. GABAergic amacrine cells are located with their somata in the INL (normal amacrine cells) and the GCL (displaced amacrine cells) [21,22]. Double labeling of vertical retinal cryostat sections from Cplx 1 KO mice with antibodies against GABA and Cplx 1/2 revealed that in the INL most of the Cplx 2-positive amacrine cells were GABA-positive. The GABA-positive/Cplx 2-positive cells accounted for ~73% (±5% SD) of the total population of GABAergic amacrine cells in the INL; this leaves ~27% (±5% SD) of the GABA-positive cells that were Cplx 2-negative (Figure 6A,A′). In the GCL, the majority of GABAergic amacrine cells were Cplx 2-positive, accounting for ~52% (±5% SD) of the population of Cplx 2-positive cells in the GCL (Figure 6A,A′).

Glycinergic amacrine cells: Glycine is the second major inhibitory neurotransmitter in the mammalian retina. Glycinergic amacrine cells are located with their somata solely in the INL [21,22]. To label glycinergic amacrine cells, we employed an antibody against the glycine transporter 1 (GlyT1), which is expressed by the entire population of glycinergic amacrine cells in the mouse retina [23] (Figure 6B). By using GlyT1 as a marker for glycinergic amacrine cells, instead of glycine, we avoided possible staining of the ON-cone bipolar cells, which have been shown to receive glycine from AII amacrine cells by diffusion through gap junctions [24]. Double labeling of vertical retinal cryostat sections from Cplx 1 KO mice with antibodies against GlyT1 and Cplx 1/2 showed that glycinergic amacrine cells were not labeled for Cplx 2 (Figure 6B,B′), corroborating the published data [4].

Cholinergic amacrine cells: Most amacrine cells are either GABAergic or glycinergic, but there are amacrine cell types, such as the cholinergic/GABAergic amacrine cell, that release more than one neurotransmitter [20,25,26,27,28]. To label cholinergic amacrine cells, which have their somata located in the INL (normal amacrine cells) and the GCL (displaced amacrine cells), we employed an antibody against choline acetyltransferase (ChAT) [16] (Figure 6C). The double labeling experiments for ChAT and Cplx 1/2 on vertical retinal cryostat sections from Cplx 1 KO mice revealed that all normal and displaced cholinergic amacrine cells were labeled for Cplx 2. In our staining approach, this accounted for 32% (±6% SD) and 22% (±6% SD) of the population of Cplx 2-positive cells in the INL and GCL, respectively (Figure 6C,C′). The processes of the cholinergic amacrine cells, which stratify in two distinct strata in the IPL [16,29], were weakly (OFF cholinergic stratum) and strongly (ON cholinergic stratum) labeled for Cplx 2 (Figure 6C).

Glutamatergic amacrine cells: The major excitatory neurotransmitter in the mammalian retina is glutamate, which is released from the ribbon synapses of the photoreceptors and bipolar cells [5]. Recently, it has been demonstrated that an amacrine cell that expresses the vesicular glutamate transporter 3 (vGluT3) also releases glutamate in addition to glycine [30,31,32,33]. We were therefore interested in finding out which of Cplx 1 to 4 is present in this special amacrine cell type. The somata of the vGluT3 amacrine cells are located in the INL and their synaptic terminals ramify between the two cholinergic strata in the IPL (Figure 7). Double labeling experiments for vGluT3 and Cplx 1/2, Cplx 3 and Cplx 4 showed that the vGluT3 amacrine cells are most likely devoid of Cplxs (Figure 7).

### 2.5. Cplx 1 and Cplx 2 Expressing Cell Types in the Ganglion Cell Layer in Mouse Retinae

**Figure 8:**
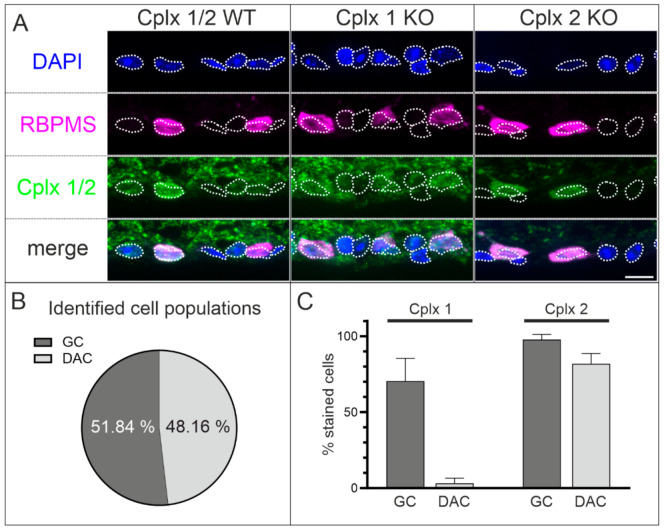
Expression of Cplx 1 and Cplx 2 in ganglion cells (GC) and displaced amacrine cells (DAC) in the ganglion cell layer (GCL) in mouse retinae. (**A**) Fluorescence micrographs of vertical cryostat sections through retinae of wildtype (WT) and Cplx 1 and Cplx 2 knockout (KO) mice triple labeled with DAPI and antibodies against the RNA-binding protein with multiple splicing (RBPMS; GC) and Cplx 1/2. White dotted lines outline DAPI-positive nuclei. (**B**) Identified cell populations of GC and DAC. (**C**) Quantification of the Cplx 1- and Cplx 2-positive GC and DAC somata. Sum of all stained cell somata = 100%; number of analyzed cells for (**B**) 521 GC and 484 DAC and (**C**) 248 GC and 247 DAC for Cplx 2 KO, and 273 GC and 237 DAC for Cplx 1 KO. Bar graphs display the mean percentages ± SD in three animals per genotype. Scale bar = 10 µm in (**A**).

The results from in situ hybridization show the presence of both Cplx 1 and Cplx 2 transcripts in cells of the GCL (Figure 2). As the GCL contains both ganglion cells and displaced amacrine cells, we wanted to find out more about the expression of the two Cplx isoforms in the population of ganglion cells compared to the population of displaced amacrine cells. We triple labeled vertical retinal cryostat sections of WT and Cplx 1 and Cplx 2 KO mice with DAPI in combination with antibodies against the RNA-binding protein with multiple splicing (RBPMS) and Cplx 1/2 (Figure 8A). DAPI labels the entirety of the cells in the GCL, and RBPMS labels the entire population of ganglion cells [34]; the difference between the two represents the population of displaced amacrine cells. In our experiments, we found that ~52% of the DAPI-labeled cells were also RBPMS positive and were thus identified as ganglion cells, while the remaining ~48% of the RBPMS-negative cells likely represent displaced amacrine cells (Figure 8B). Within the ganglion cell population, 70% (±15% SD) and 98% (±3% SD) of the ganglion cells expressed Cplx 1 and Cplx 2, respectively; within the displaced amacrine cell population the percentages were 3% (±3% SD) for Cplx 1 and 82% (±7% SD) for Cplx 2 (Figure 8C).

**Figure 9:**
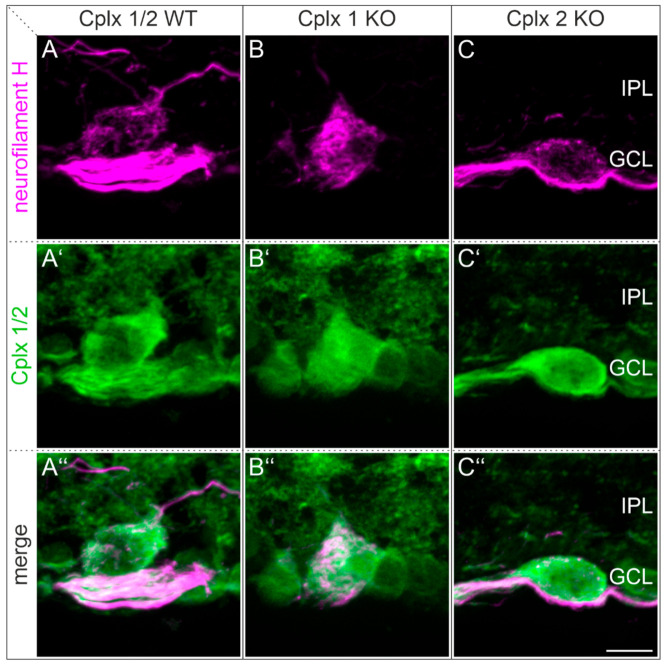
Expression of Cplx 1/2 in alpha ganglion cells in mouse retina. (**A**–**C″**) Fluorescence micrographs of vertical cryostat sections through retinae of wildtype (WT) and Cplx 1 and Cplx 2 knockout (KO) mice double labeled with antibodies against neurofilament H (alpha ganglion cells) and Cplx 1/2. IPL, inner plexiform layer; GCL, ganglion cell layer. Scale bar = 10 µm in (**C″**) for (**A**–**C″**).

In a final set of immunocytochemical experiments, we focused on alpha ganglion cells, a distinct type of retinal ganglion cell in the mammalian retina with four known subtypes in mouse retinae [35]. Alpha ganglion cells can be identified by neurofilament labeling [36,37]. We double labeled vertical retinal cryostat sections of WT and Cplx 1 KO and Cplx 2 KO mice with antibodies against neurofilament H (SMI-32) and Cplx 1/2. In the Cplx 1 and Cplx 2 KO retinae, neurofilament H-labeled alpha ganglion cells expressed Cplx 2 or Cplx 1, respectively, suggesting that alpha ganglion cells express both Cplx isoforms (Figure 9).

### 2.6. Bioinformatics Analysis of the Cell Types Expressing the Genes Encoding Cplx 1 and Cplx 2 in Mouse Retina

**Figure 10:**
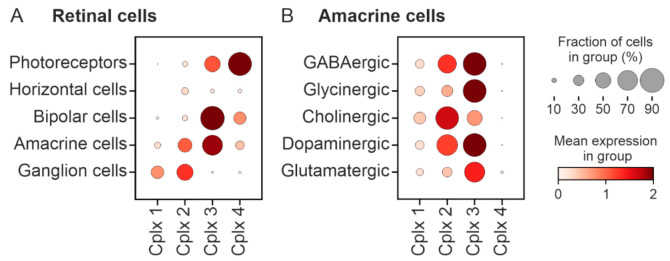
Bioinformatics analysis of the expression of *cplx 1* to *4* in cells of mouse retinae. (**A**,**B**) Clustering according to cell types (**A**) and neurotransmitter types (**B**). Visualized data are based on scRNA-seq data sets [38] (**A**) and [20] (**B**).

To further clarify the immunocytochemical results on the presence of Cplxs 1 and 2 in different retinal cell types, we performed a bioinformatics analysis of the genes, *cplx 1*, *2*, *3* and *4*, encoding all four Cplx isoforms in the cells of mouse retina. For this, we used two different scRNA-seq data sets [20,38]. With the dataset of Macosko et al. [38], retinal cell types, including photoreceptors, horizontal cells, bipolar cells, amacrine cells and ganglion cells, were investigated for their expression of *cplx 1* to *4* (Figure 10A). Based on their mean expression levels, *cplx 1* and *cplx 2* seem to be predominantly expressed in amacrine cells and ganglion cells, whereby *cplx 2* is the dominant isoform in both cell types, while *cplx 1* is mainly found in ganglion cells (Figure 10A). *Cplx 3* is expressed in photoreceptors, bipolar cells and amacrine cells, while *cplx 4* is mainly found in photoreceptors and a small population of bipolar cells (Figure 10A). Figure 10B shows a more in depth analysis of amacrine cells based on the scRNA-seq and postprocessed data set of Yan et al. [20]. Amacrine cells were clustered according to their neurotransmitter type. High expression values of *cplx 2* were found in GABAergic, cholinergic and dopaminergic amacrine cells (Figure 10B). Especially in cholinergic amacrine cells, the fraction of cells expressing *cplx 2* was almost 100%, confirming the results from immunocytochemistry (Figure 6). An unexpected result from the bioinformatics analysis was the expression of *cplx 3* in all amacrine cell types (Figure 10B), which is not in accordance with immunocytochemistry. In contrast to this, the absence of *cplx 4* expression from amacrine cells agrees well with the results from immunocytochemistry [4] (Figure 10B).

In summary, the results from immunocytochemistry and bioinformatics (Figure 6, Figure 7, Figure 8, Figure 9 and Figure 10) convincingly demonstrate that Cplx 2 and not Cplx 1 is the main Cplx isoform of normal and displaced amacrine cells and of ganglion cells in mouse retinae.

### 2.7. Postnatal Developmental Expression of the Cplxs 1 to 4 in Mouse Retina

**Figure 11:**
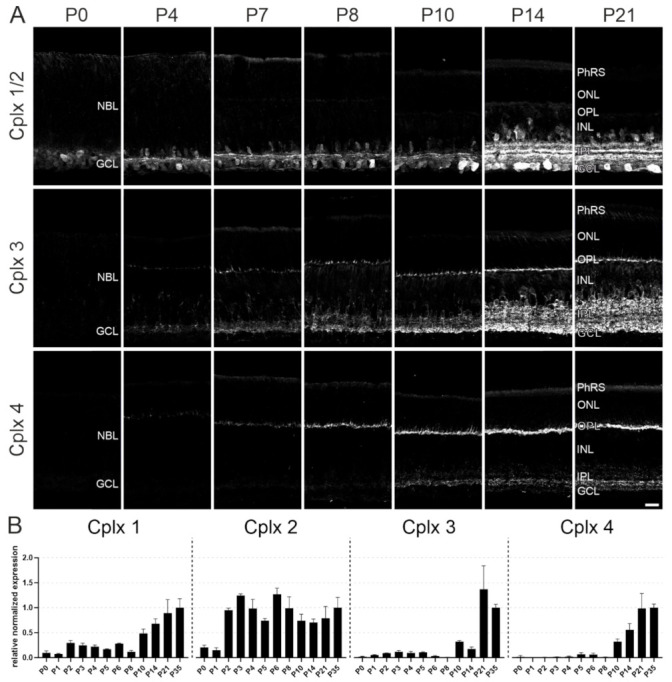
Postnatal developmental expression of Cplxs 1–4 in mouse retinae. (**A**) Fluorescence micrographs of vertical cryostat sections through retinae of wildtype (WT) mice at different postnatal developmental ages stained with antibodies against Cplx 1/2, Cplx 3 and Cplx 4. (**B**) RT-qPCR analysis of Cplxs 1–4 expression in WT retinae at different postnatal developmental ages. Bar graphs display the mean relative normalized expression levels ± SD in three animals per age group. P, postnatal day; NBL, neuroblast layer; PhRS, photoreceptor segments; ONL, outer nuclear layer; OPL, outer plexiform layer; INL, inner nuclear layer; IPL, inner plexiform layer; GCL, ganglion cell layer. Scale bar = 20 µm in (**A**).

In mouse retina, all four known members of the Cplx family are present [4]. In a final set of experiments, we examined the expression of the Cplx isoforms during early postnatal retinal synaptogenesis by staining vertical cryostat sections from P0, P4, P7, P8, P10, P14 and P21 WT mouse retinae with antibodies against Cplx 1/2, Cplx 3 and Cplx 4 (Figure 11A). To be able to better compare the results, we used the same antibody solutions for the respective Cplx isoforms for all examined time points, and we took all images with the identical exposure time and adjusted the images equally for brightness and contrast (Figure 11A). Finally, we performed an even more detailed temporal analysis of the mRNA expression of the four Cplxs with RT-qPCR, which also allowed us to distinguish between Cplx 1 and Cplx 2 (Figure 11B).

Cplxs 1 and 2 were the first Cplxs to be detected in the postnatal developing retina (protein and transcript). Already at P0, weak Cplx 1/2 immunoreactivity was present in the region of the forming IPL/GCL (Figure 11). Staining intensity increased gradually from P0 to P14, when Cplx 1/2 immunoreactivity reached its mature staining pattern with labeled somata of amacrine cells, displaced amacrine cells and ganglion cells in the INL and the GCL and three labeled strata in the IPL (Figure 11).

Cplx 3 was first detected at P4, in a very weakly immunoreactive thin layer in the undifferentiated neuroblast layer (NBL) at the future forming OPL and also in the IPL (Figure 11). Cplx 3 immunoreactivity increased gradually from P4 to P21, when Cplx 3 reached its mature staining pattern with strong immunoreactivity in both plexiform layers of the retina, the OPL and IPL, and with labeled amacrine cell somata in the INL (Figure 11).

Cplx 4, like Cplx 3, was first detected at P4 in the NBL in a thin, very weakly immunoreactive band at the future-forming OPL (Figure 11). The first weak Cplx 4 immunoreactivity in the IPL appeared later in postnatal retinal development around P10 (Figure 11). At P21, Cplx 4 immunoreactivity reached its mature labeling pattern, with strong immunoreactivity in the OPL and a broader immunoreactive band approximately in the middle of the IPL (Figure 11).

In summary, the temporal sequence (protein and transcript) in the appearance of Cplxs 1 and 2 followed by Cplxs 3 and 4 corresponds to the slightly different developmental time course of conventional and ribbon synapses in mouse retina. Conventional synapses in the IPL appear around postnatal day 3 (P3) [39,40] and ribbon synapses in the OPL and IPL appear around P4 [41,42] and P11 [39], respectively.

## 3. Discussion

Cplxs are important SNARE complex regulators, which determine the speed and accuracy of exocytotic fusion of synaptic vesicles (for review see [2,43,44]). We showed previously that all four known Cplxs, Cplxs 1 to 4, are present in mouse retina, whereby Cplxs 1 and 2 seem to be restricted to conventional chemical synapses and absent from ribbon synapses [4]. Here we analyzed in detail the cell types and synapses that express these two Cplx isoforms in mouse retinae.

### 3.1. Presence of Cplx 2 but Not Cplx 1 at Conventional Chemical Synapses in Mouse Retinae

First, we examined if Cplx 1 or Cplx 2 deficiency compromised gross retinal anatomy and neuronal morphology, which could distort the results from the immunocytochemical analyses. The two Cplx KO mouse lines showed no overt morphological retinal phenotype (Figure 3). Unexpected was the functional ERG phenotype in the Cplx 1 KO mice with (i) an additional shoulder riding on the peak of the scotopic b-wave, resulting in an apparent increased delay of the b-wave; and (ii) an additional positive peak in the descending flank of both the scotopic and photopic b-wave (Figure 5). This second positive peak occurs very late, indicating that its cellular origin differs from that of the b-wave. It superficially resembles the i-wave recorded in other species; however, an i-wave is absent in the mouse ERG [45]. Furthermore, the i-wave is mainly found in photopic ERG, whereas we recorded the positive peak also under scotopic conditions (Figure 5). Possibly, the response originates from light-induced muscle contractions. In contrast to Cplx 2 KO mice, Cplx 1 KO mice show severe motoric deficits and suffer from strong ataxia and sporadic seizures [14,43,46]. However, the strong synchronization with the stimulus is indicative for a retinal origin. The late occurrence of the second positive peak would argue for an inner retinal origin, which would also be in agreement with our finding that Cplx 1 is mainly detected in retinal ganglion cells (Figure 2, Figure 8 and Figure 10). Perhaps, altered feedback mechanisms from ganglion cells to amacrine and/or bipolar cells may be responsible for this component. We are not aware of comparable components described in the literature, and further studies are necessary to draw more definite conclusions about its physiological origin.

A key result of our study is the presence of Cplx 2 and the most likely absence of Cplx 1 in conventional chemical synapses of normal and displaced amacrine cells (Figure 2, Figure 6, Figure 8 and Figure 10). Cplx 2 (protein and mRNA) was found in GABAergic, cholinergic and dopaminergic amacrine cells but not in glycinergic amacrine cells (Figure 6 and Figure 10), which contain Cplx 3 [4]. The glutamatergic vGluT3 amacrine cells also contained no Cplx 2 (Figure 7 and Figure 10). VGluT3 amacrine cells represent an unconventional type of amacrine cell with dual release of glycine and glutamate at segregated synaptic sites. It is suggested that the release of an inhibitory and an excitatory neurotransmitter into two separate neuronal circuits by the vGluT3 amacrine cells facilitates differential detection of visual field uniformity and contrast [33,47]. As Cplx 3 is found in glycinergic amacrine cells and glutamatergic rod bipolar cells [4,48], we analyzed its presence in vGluT3 amacrine cells. We could not detect Cplx 3 in these retinal interneurons, just as we could not detect Cplx 4 in vGluT3 amacrine cells (Figure 7). The absence of Cplxs 1 to 4 from vGluT3 amacrine cells suggests a Cplx-independent release of neurotransmitters. Likewise, for the auditory sensory cells of the cochlea, the inner hair cells, it has been suggested that they release glutamate at their ribbon synapses independent of neuronal SNARE proteins [49] and SNARE regulators, such as Cplxs and synaptotagmins [50,51,52].

Taken together, with the exception of vGluT3 amacrine cells, the main amacrine cell types in the mouse retina seem to operate with a single Cplx isoform at their conventional chemical synapses. This is in contrast to brain neurons that are often equipped with more than one Cplx isoform; for example, pyramidal cells and granule cells in the hippocampus and granule cells and Purkinje cells in the cerebellum [53,54,55]. Amacrine cells are the most heterogeneous class of retinal interneurons with more than sixty different types [20]. As each amacrine cell type fulfills a specific role in the processing of visual signals, the presence of a given Cplx isoform at their synapses might contribute to shaping the response of ganglion cells to visual signals and thus the output of the retina to the brain [20]. The concept of specificity of the presynaptic molecular machinery applies not only to the Cplxs but also to the Munc13s, which operate as single isoforms at amacrine cell synapses [56].

Another class of retinal interneurons are horizontal cells. By their interaction with photoreceptors and bipolar cells in the outer retina, they generate receptive field surrounds and enhance spatial discrimination. Vesicular release of GABA is among the proposed mechanisms for horizontal cell feedback to photoreceptors [57,58]. We showed in previous [4,59] and in this study that horizontal cells in mouse and rabbit retina express Cplxs 1 and 2. The results of our bioinformatics analysis indicate that Cplx 2 is the isoform expressed in mouse retinal horizontal cells (Figure 10).

### 3.2. Presence of Cplx 1 and Cplx 2 in Ganglion Cells in Mouse Retina

The ganglion cells are the sole output neurons of the retina. To date, more than 40 ganglion cell types are known that encode different visual features and transmit this information in parallel channels to the different visual centers of the brain [60,61,62,63,64]. It was surprising to find Cplx 1 and Cplx 2 in ganglion cells (Figure 8 and Figure 10), and the results even suggest that alpha ganglion cells may express both Cplx isoforms (Figure 9). We currently have no idea what a dual expression of Cplxs 1 and 2 in ganglion cells could mean functionally. Nevertheless, our data on amacrine cells suggest that Cplxs 1 and 2 are not simply two highly homologous proteins with functional redundancy but that their selective synaptic expression reflects a specific function in the processing of visual signals.

Similarly puzzling is the distinct labeling of ganglion cell dendrites for Cplxs 1 and 2 in the IPL (Figure 1, Figure 4 and Figure 9). Ganglion cells branch with their dendrites in different strata in the IPL, where they receive excitatory and inhibitory synaptic inputs from bipolar and amacrine cells, respectively. Dendrodendritic electrical coupling of homotypic and heterotypic ganglion cells in mammalian retinae has been described [65,66]; but, to our knowledge, no reciprocal chemical synapses exist between the dendrites of ganglion cells and processes of bipolar and amacrine cells. This leaves us with a postsynaptic localization of Cplxs 1 and 2 in ganglion cell dendrites in the mouse retina and the possibility that Cplx 1/2-deficiency could affect both pre- and postsynaptic function in the retina. Postsynaptic labeling for Cplx has been described in dendritic spines and shafts and postsynaptic densities in the cerebral cortex [3,54] and in principal neurons of the medial nucleus of the trapezoid body [67], and postsynaptic Cplx has been shown to be essential for AMPA receptor exocytosis during LTP in hippocampal CA1 pyramidal cells [68].

In conclusion, the cell type and synapse-specific distribution of Cplxs 1 and 2 in the mammalian retina is further proof of the intricate specificity and heterogeneity of pre- and postsynaptic machineries, contributing to the complexity of visual signal processing in mammalian retinae.

## 4. Materials and Methods

### 4.1. Animals

Animal experiments were approved by the local authorities (Regierung von Mittelfranken, AZ 54.2531.31_26/07; Amt für Veterinärwesen der Stadt Erlangen, AZ TS10/07) and conducted in accordance with the European Communities Council Directive (2010/63/EU). For developmental studies, C57BL/6J mice at the indicated ages were used. Otherwise, adult (2–4 months) C57BL/6J or Cplx 1 and Cplx 2 KO mice were used. Cplx 1 and Cplx 2 KO mice were provided by Dr. Kerstin Reim, Max-Planck-Institute for Experimental Medicine, Göttingen, Germany [AZ 33.9-42502-04-15/1921 (2015–2020); AZ 33.19-42502-04-20/3518 (2020–2023)]. Animals were housed in a 12h/12h light/dark cycle with food and water provided ad libitum.

### 4.2. Antibodies

The following primary antibodies were used for immunocytochemistry: rabbit anti-CaBP5 (1:500; [17]), rabbit anti-calbindin D28k (1:1000; Swant, Marly, Switzerland, cat. no. CB-38), mouse anti-calretinin (1:2000; Chemicon, Temecula, CA, USA, cat. no. MAB1568), goat anti-glycine transporter 1 (1:10,000; Chemicon, cat. no. AB1770), goat anti-ChAT (1:400; Chemicon, cat. no. AB144P), rabbit anti-complexin 1/2 (1:10,000; Synaptic Systems GmbH, Göttingen, Germany, cat. no. 122 102), rabbit anti-complexin 3 (1:10,000; Synaptic Systems GmbH, cat. no. 122 302), rabbit anti-complexin 4 (1:40,000; Synaptic Systems GmbH, cat. no. 122 402), guinea pig anti-complexin 1/2 (1:50,000; Synaptic Systems GmbH, cat. no. 122 104), guinea pig anti-vesicular glutamate transporter 3 (1:3000; Synaptic Systems GmbH, cat. no. 135 204), rabbit anti-GABA (1:500; Sigma-Aldrich, Merck KGaA, Darmstadt, Germany, cat. no. A2052), mouse anti-SMI-32 (1:1000; Biolegend, San Diego, CA, USA, cat. no. 80170) and rabbit anti-RBPMS (1:500; Thermo Fisher Scientific, Waltham, MA, USA, cat. no. PA5-31231). Fluorophore-coupled secondary antibodies were used for visualization of the primary antibodies: Alexa^®^ 488/568-conjugated goat anti-mouse, goat anti-rabbit and goat anti-guinea pig IgG (1:500; Thermo Fisher Scientific, cat. nos. A-11001, A-11004, A-11034, A-11011, A-11075, A-11073), Alexa^®^ 488-conjugated donkey anti-rabbit IgG and Alexa^®^ 568-conjugated donkey anti-goat IgG (1:500; Thermo Fisher Scientific, cat. nos. A-21206, A-11057). Cell nuclei were labelled with DAPI (0.1 µg/mL), and the cone photoreceptor outer segments and synapses were stained with rhodamine-coupled peanut agglutinin (1:500; Vector Laboratories, Burlingame, CA, USA, cat. no. RL-1072-5).

### 4.3. Immunocytochemistry

Preparation of the retinae of male and female mice and antibody incubation for light microscopic immunocytochemistry were done as described previously with minor modifications [56]. Briefly, mice were deeply anesthetized by inhalation of isoflurane (Abbott Laboratories, Chicago, IL, USA) and killed by cervical dislocation. For cryostat sections, the eyes were opened and fixed in the eyecup in 4% paraformaldehyde (PFA) in PBS (0.01 mol L^−1^, pH 7.4) for 30 min (Figure 3 and Figure 6, Figure 7, Figure 8, Figure 9) or 60 min (Figure 11) at room temperature (RT). After washing, the retinae were cryoprotected in 10%, 20% and 30% (*w*/*v*) sucrose in 0.01 mol L^−1^ PBS, dissected free and mounted in Tissue-Tek O.C.T. freezing medium (Sakura Finetek Germany, Staufen, Germany). Vertical cryostat sections (14 μm thick) were cut with a cryostat (CM3050 S, Leica Microsystems, Wetzlar, Germany). Sandwiches of WT and Cplx 1 and Cplx 2 KO (littermates) retinae were collected on the same slides to ensure equal immunocytochemical treatment. Cryostat sections were washed in 0.01 mol L^−1^ PBS and blocked in blocking solution (10% normal goat or donkey serum (NGS/NDS), 1% bovine serum albumin (BSA), 0.5% Triton X-100 in 0.01 mol L^−1^ PBS) for 1 h at RT. Primary antibodies were diluted in antibody solution (3% NGS/NDS, 1% BSA, 0.5% Triton X-100 in 0.01 mol L^−1^ PBS) and incubation was performed overnight at 4 °C. The next day, sections were washed with 0.01 mol L^−1^ PBS and incubated with secondary antibodies diluted in antibody solution for 1 h at RT. After washing, samples were mounted in Aqua Polymount (Polysciences, Warrington, PA, USA).

For vibratome sections, the eyes were opened and fixed in an eyecup in 4% PFA and 0.02% picric acid in a phosphate buffer (PB, 0.1 mol L^−1^, pH 7.4) for 30 min. After washing and cryoprotection as described above, retinae were cracked three times using a liquid nitrogen-cooled copper block. Retinae were embedded in 3% low melting agarose and cut with a vibratome (VT1000S, Leica Microsystems) to obtain 60 µm-thick vertical sections. Immunocytochemistry with floating vibratome sections was performed as described above for cryostat sections with blocking, primary antibody incubation and secondary antibody incubation for 90 min, 3 days and 3 h, respectively. After the final washing steps, the samples were mounted on glass slides using Aqua Polymount (Polysciences).

### 4.4. Light Microscopy and Analysis of Immunocytochemical Data

For analysis, the labeled sections were examined with an Axio Imager.M2 equipped with an ApoTome.2 module or a Laser Scanning Microscope 710 with corresponding imaging modules (Carl Zeiss AG, Oberkochen, Germany). Images were acquired using a 20 × (0.8 NA, Apochromat) or a 63 × (1.4 NA oil immersion, Plan Apochromat) objective (both Carl Zeiss AG) as stacks of multiple optical sections and projections were calculated with ZEN blue or ZEN black software (Carl Zeiss AG). Images were adjusted for contrast and brightness using Photoshop CS6 (Adobe Systems, San Jose, CA, USA) and arranged using CorelDRAW (Corel Corporation, Ottawa, ON, Canada). For quantifying the colocalization experiments, three animals of each genotype were analyzed. Per animal, three images were acquired at different regions of the retina and cell counting was performed using ZEN blue (Carl Zeiss AG). Cell numbers were summarized in Excel (Microsoft Corporation, Redmond, WA, USA) and graphs were created using GraphPad Prism 9.0 software (GraphPad Software Inc., San Diego, CA, USA). Graphs and images were arranged using CorelDRAW (Corel Corporation).

### 4.5. Electroretinographic (ERG) Recordings and Data Analysis

The mice were dark adapted overnight, and all further handling was performed under deep red illumination to maintain the dark adaptation. The mice were anesthetized with an intramuscular injection of 50 mg kg^−1^ ketamine (Ketavet^®^, Pfizer, Deutschland GmbH, Berlin, Germany) and 10 mg kg^−1^ xylazine (Rompun^®^ 2%, Bayer HealthCare AG, Leverkusen, Germany). A subcutaneous injection of saline solution (300 µL, 0.9%) was given to protect the mice from dehydration. Pupils were dilated with a drop of tropicamide (Mydriaticum Stulln^®^, 5 mg mL^−1^, Pharma Stulln GmbH, Stulln, Germany) and phenylephrin-hydrochloride (Neosynephrin POS^®^ 5%, Ursapharm Arzneimittel GmbH, Saarbrücken, Germany). Anesthetized mice were placed on a heated table to maintain body temperature during ERG recordings. To measure the ERG, the ground needle electrode was placed subcutaneously at the base of the tail, the reference needle electrodes were positioned subcutaneously, medially to the ears, and the active contact lens electrodes (diameter 3.2 mm) (Mayo Corporation, Takamido, Inazawa, Aichi, Japan), internally covered with Corneregel^®^ (Dr. Mann Pharma, Berlin, Germany) to improve electrical contact and avoid dehydration of the cornea, were placed on the cornea of each eye. To deliver the stimuli, a Ganzfeld Bowl (Q450 SC, Roland Consult, Brandenburg a. d. Havel, Germany) was used. Stimulation and data recording were controlled using the RetiPort system (Roland Consult). Scotopic flash ERGs were recorded with the flash strength increasing in eight steps (−3.7, −2.7, −2.2, −1.7, −1.2, −0.7, −0.2 and 0.8 log cd × s/m^2^) and, depending on flash strength, 8 to 12 flashes were averaged. Flash duration varied between 5 µs and 5 ms depending on the required total energy. Stimulation frequency increased with increasing flash strength from 1 to 0.05 Hz and the time between two intensity steps increased from 10 to 120 s. After adapting the mice for five minutes to a 1.4 log cd/m^2^ steady background light, photopic flash ERGs with a 0.8 log cd × s/m^2^ flash strength were recorded. In total, 20 sweeps presented at 1 Hz were averaged. ERG signals were amplified 100,000 times, band-pass filtered between 1 and 300 Hz and digitized with a sampling frequency of 2048 Hz.

The ERG recordings were analyzed with custom-written MATLAB programs (The Math Works Inc., Natick, MA, USA). ERG traces were Fourier transformed to separate the a- and b-wave components from the oscillatory potentials by dividing the frequency spectrum in a low and high frequency range [69]. The inverse Fourier transform of the lower frequency range was used to obtain the amplitudes and latencies of the a- and b-wave. The maximal amplitude of the higher frequency range of the Fourier transform was used to quantify the amplitude of the oscillatory potentials [66]. Figure 5 displays the group averages of the original, i.e., unprocessed, responses. The a-wave amplitude was defined from baseline to the a-wave trough, and the b-wave amplitude was measured from the trough of the a-wave to the peak of the b-wave. Latencies of the a- and b-wave were defined as the time difference between flash onset and time of occurrence of the a-wave trough or the b-wave peak. Wildtype littermates of the Cplx 1 and Cplx 2 knockout mice were pooled as one wildtype group with a total *n* of 16. For statistical analyses, unpaired *t*-tests were performed in SPSS (Version 24, IBM, Armonk, NY, USA) after testing for a Gaussian distribution. An alpha-value of 0.05 was adopted for statistical significance. The results were Bonferroni corrected for multiple testing.

### 4.6. In Situ Hybridization

In situ hybridization was performed as previously described [48]. Probes for Cplx 1 and Cplx 2 were generated by polymerase chain reaction (PCR) using genomic mouse wildtype DNA as template. The resulting fragments (for *cplx 1*, bp 624–844 of NM_007756, for *cplx 2* bp 867–1102 of NM_001362218) were directly cloned into the pCRII-TOPO vector of the TOPO-PCR-Cloning Kit (Invitrogen, Karlsruhe, Germany), in accordance with the manufacturer’s instructions.

### 4.7. RT-qPCR

Freshly isolated retina tissue was homogenized in RLT buffer (Qiagen, Hilden, Germany) containing 1% β-mercaptoethanol. Total RNA was isolated using the RNeasy Mini Kit (Qiagen) and cDNA synthesis (reverse transcriptase reaction) was performed using the iScript cDNA Synthesis Kit (Bio-Rad Laboratories, Munich, Germany). RT-qPCR was performed with a 59 °C annealing temperature in a volume of 12.5 µL using 1 µL of the prepared cDNA and 0.3 µL of each primer (of the 10 pm working solution)/reaction. For sequence comparisons and oligonucleotide generation, the computer program SnapGene 4.0.8 (GSL Biotech LLC, San Diego, CA, USA) was used. Gene expression was normalized to actin, GAPDH and PBGD and quantified using CFX Manager 3.1 Software (Bio-Rad Laboratories) and Excel (Microsoft Corporation). The graphs were created using GraphPad Prism 9.0 software (GraphPad Software Inc.). The specific primer pairs are summarized in Table 1.

### 4.8. Bioinformatics

We used the datasets of Macosko et al. [38] and Yan et al. [20] for a bioinformatics analysis of the genes encoding Cplx 1 (*cplx 1*) and Cplx 2 (*cplx 2*) in different cell types in mouse retinae. The dataset of Macosko et al. [38] provides single-cell sequencing results for the complete mouse retina and its distinct cell types. For the analysis, we used the clustering as published and the extracted cells that have been assigned as retinal ganglion cells, amacrine cells, horizontal cells, bipolar cells and photoreceptors. In the latter, we combined rod and cone photoreceptors. The different cell types were tested for their mean expression of *cplx 1* and *cplx 2*. To analyze the dataset of Yan et al. [20], the cluster file was used to obtain the clusters as published. These clusters were evaluated according to their gene expression profile and assigned to a neurotransmitter type. As the marker genes we used *gad1* and *gad 2* (GABA), *slc6a9* and *slc6a5* (glycine), *slc17a8* (glutamate), *chat* (choline) and *Th* (dopamine). If the respective cluster showed a clear expression of the particular marker gene, it was assigned to the corresponding neurotransmitter type. This assignment was not exclusive. A cluster could be assigned to more than one neurotransmitter type. The evaluation was done manually. The resulting groups were finally tested for their expression of *cplx 1* and *cplx 2* and visualized. Graphs and the preceding analysis were performed using Python and in particular the Python toolkit Scanpy v1.7.2 [70].

## Figures and Tables

**Table 1:** Specific primer pairs for RT-qPCR (5′–3′).

	Forward	Reverse
Cplx 1	GATCGTCCACCTTGGAAG	GATGGCTTGGTTCCTCAG
Cplx 2	AGTGGCTTAGACGGTTG	TGCAGGCTTTGGTTAATG
Cplx 3	GAAGAGTACGAGGAGTATC	CTTCCTCTGTGTGAACTG
Cplx 4	GGCTAAAGGGATGACTAG	CTCTCTCCATCTTCTCTTC
Actin	TTCCTCCCTGGAGAAGAG	CACTGTGTTGGCATAGAG
GAPDH	CAACTTTGTCAAGCTCATT	TCTGGGATGGAAATTGTG
PBGD	TACCCTGGCATACAGTTT	CGTTTTCTAGCTCCTTGG

## Data Availability

For the analysis of the dataset of Macosko et al. [38], we used the clustering as published and available at https://singlecell.broadinstitute.org/single_cell/study/SCP7/drop-seq (accessed on 12 February 2021). To analyze the dataset of Yan et al. [20] available at https://singlecell.broadinstitute.org/single_cell/study/SCP919/mouse-retinal-cell-atlas-molecular-identification-of-over-sixty-amacrine-cell-types (accessed on 2 March 2021), the cluster file was used to obtain the clusters as published by Yan et al. [20].

## Abbreviations

AC	amacrine cell
DAC	displaced amacrine cell
GC	ganglion cell
GCL	ganglion cell layer
INL	inner nuclear layer
IPL	inner plexiform layer
KO	knock-out
ONL	outer nuclear layer
OP	oscillatory potentials
OPL	outer plexiform layer
PhRS	photoreceptor segments
